# Supplementary material for: A role for astrocytic miR-129-5p in frontotemporal dementia
Source: Transl Psychiatry. 2025 Apr 11;15:142. doi: 10.1038/s41398-025-03338-y (PMC11992244; doi:10.1038/s41398-025-03338-y)
Supplement: Supplementary file 1 — Supplemental figures [file 41398_2025_3338_MOESM1_ESM.pdf]

Supplemental Figure 1 (Fig S1).

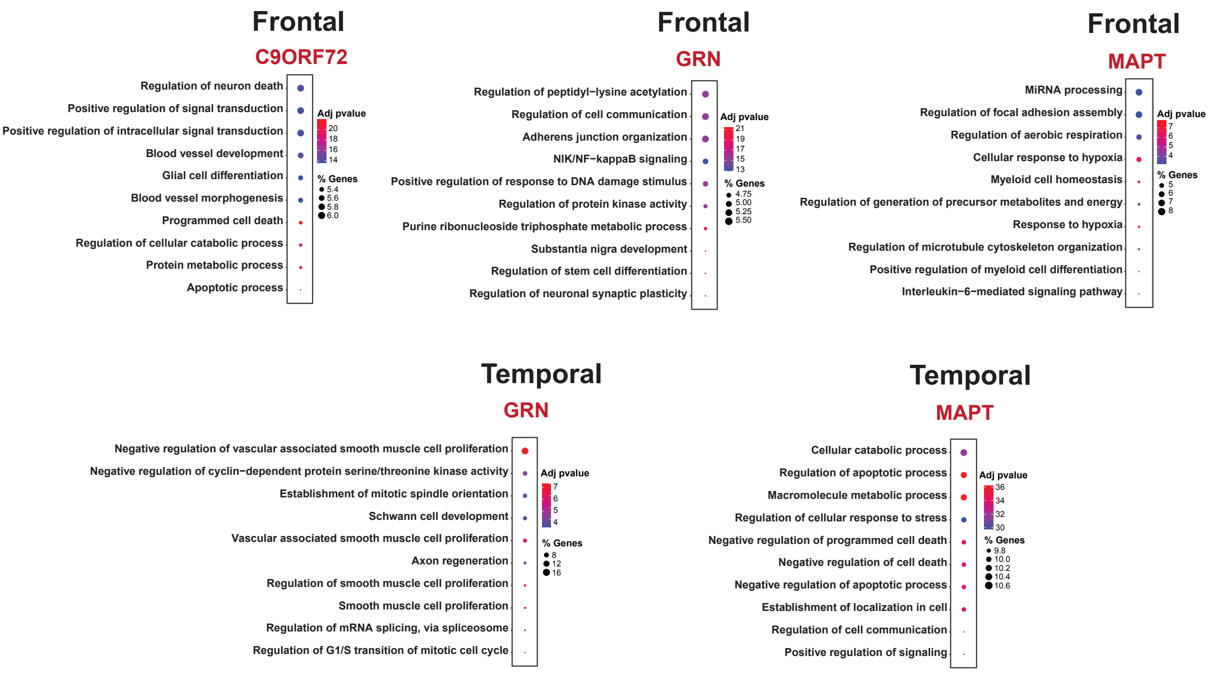

Fig. S1. Pathways Affected by miRNAs Specifically Deregulated in Response to either *C9ORF72*, *GNR* or *MAPT* mutations and the frontal or temporal lobe. The graphs display the top 10 enriched GO terms (Biological processes) identified based on brain-expressed target transcripts of miRNAs deregulated exclusively in the depicted condition. It's important to note that no GO term analysis was conducted for the temporal lobe in the case of *C9ORF72* carriers, as only one miRNA was deregulated in this condition

Supplemental Figure 2 (Fig S2).

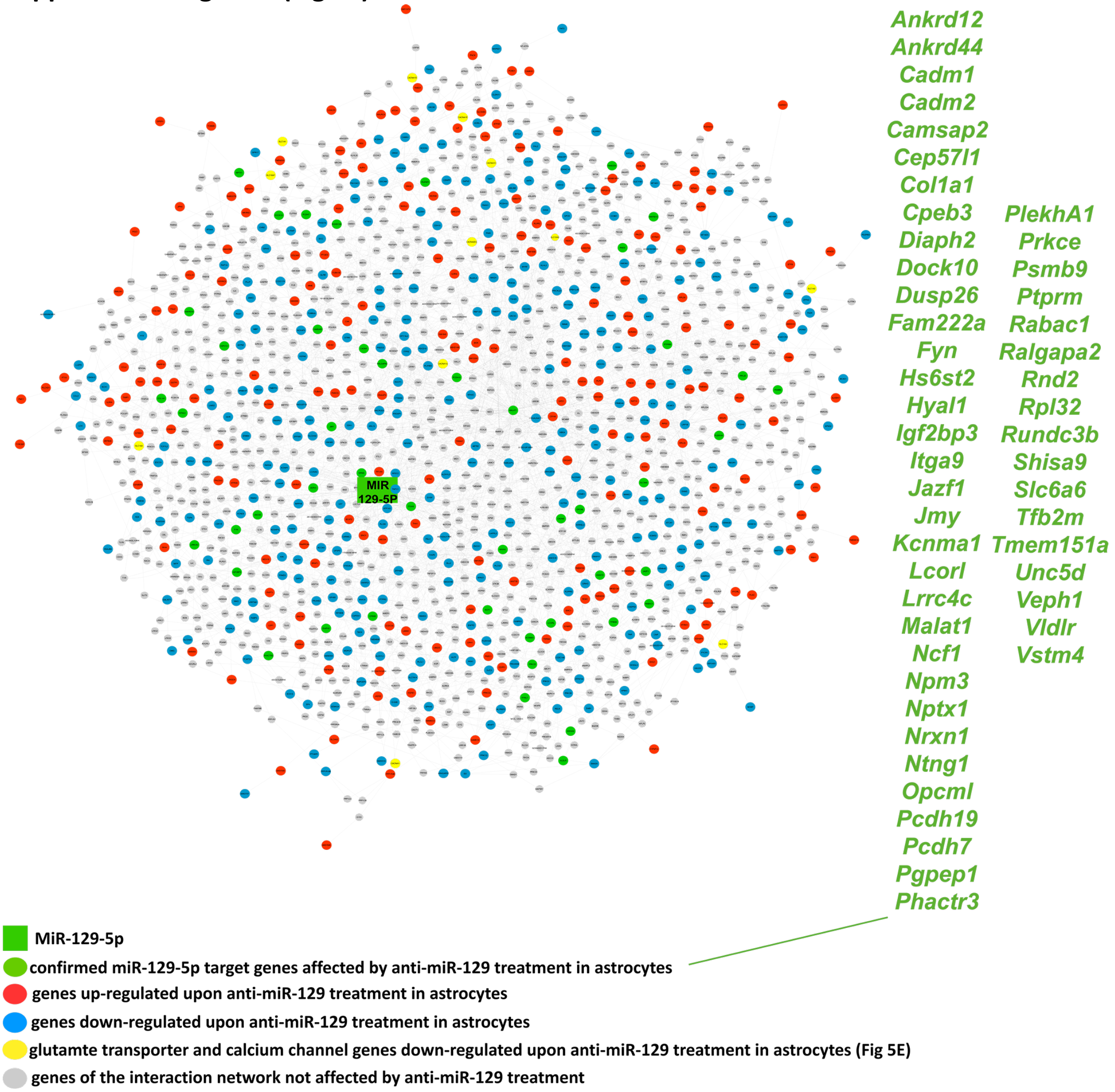

Fig. S2. mIR-129-5p interaction network. We identified 50 confirmed miR-129-5p target genes (shown in green) among the transcripts deregulated in astrocytes upon miR-129-5p knockdown. Using these data, we built a gene expression interaction network that could explain approximately 80% of the transcripts detected as differentially expressed in the corresponding RNA-seq data. In yellow, we highlight the genes that were downregulated upon miR-129-5p knockdown and encode glutamate transporters and calcium channels, confirmed via qPCR (see Fig. 5E).
